# Supplementary material for: Molecular dissection of the soluble photosynthetic antenna from the cryptophyte alga Hemiselmis andersenii
Source: Commun Biol. 2023 Nov 13;6:1158. doi: 10.1038/s42003-023-05508-4 (PMC10643455; doi:10.1038/s42003-023-05508-4)
Supplement: Supplementary file 2 — Supplementary Information [file 42003_2023_5508_MOESM2_ESM.pdf]

Molecular dissection of the soluble photosynthetic antenna from the cryptophyte alga *Hemiselmis andersenii*

Authors

Harry W. Rathbone[1,2], Alistair J. Laos[3], Katharine A. Michie[1,2,4], Hasti Iranmanesh[1,2], Joanna Biazik[4], Sophia Goodchild[5], Pall Thordarson[3], Beverley R. Green[6] and Paul M. G. Curmi[1,2]*

Supplementary Information

Supplementary Notes

Supplementary Note 1 —Unclassified peaks in chromatography

Three peaks in the chromatography did not appear to fit a specific spectrotype when examining their absorption spectra (Supplementary Fig. 11). These were labelled XA, XB and XC. Each spectrum had features of multiple spectrotypes indicating that they are mixtures possibly due to lack of resolution between peaks or contamination from larger neighboring peaks. Furthermore, the mass spectrometry for fraction XA has, amongst masses for *Ha*PE555 type proteins, a mass for HA$\alpha^{C}{}_{1}$/HA$\alpha^{C}{}_{2}$ and the spectrum has a minor peak at 645 nm suggesting that a *closed* form (*Ha*PE645 type) protein makes up some of the protein components within this peak and producing the small hump at 645 nm. Mass spectrometry shows that *Ha*$\alpha^{OB}{}_{1}$ is also seen in the purple fraction suggesting there is some contamination arising from when pink and purple were split after the initial anion exchange chromatography.

Supplementary Note 2 —*Ha*PE555 from the smaller peak 555B

In all *Ha*PE555 structures, we observed Phe30 of the β subunit making close contacts between adjacent filaments forming 2D sheets (Supplementary Fig. 3). In the structure derived from chromatography peak 555B (8EL6; Fig. 1b), there is an alteration in the secondary structure of the β subunit around Phe30 with a lengthening helix hA and shortening hY altering the interfilament interaction (Supplementary Fig. 3bc). This reorganization of secondary structure has not been observed in any other structure of a cryptophyte PBP. This alteration is only observed for one of the two β subunits in this PBP. Given that this peak 555B separates from 555A, this suggests that this alteration in secondary structure is stable and possibly the cause of this separation. This may therefore imply that chromatography peak 555C has this alteration on both β-subunits.

Supplementary Note 3 — *Ha*PE555 filaments

All five crystal forms of protein *Ha*PE555 are constructed from continuous filaments of PBPs (Supplementary Fig. 2b). The number of PBPs per asymmetric unit varies, either one (8EL4 and 8EL6), two (8EL3 and 8EL5) or three (4LMX). The filament structures observed in the five different crystals are generated from knobs-within-holes packing but differ from each other in the distances between adjacent molecules along the filament direction (having either tight or loose interfaces; Supplementary Fig. 2b; Supplementary Table 3). The knobs-within-holes contact is formed by the CD-loop of one β subunit slotting into a hole formed around the GH loop on an opposite β subunit (Supplementary Fig. 2b, left and right panels for tight and loose interfaces, respectively). The cleft along this interface tightens in different conditions, where a layer of waters is removed in each step of shortening. The packing changes along the filament direction are evaluated by the average period along the filament, |**c**|/molecule, which is measured by length of the unit cell vector, |**c**| (which is parallel to the filament axis), divided by number of molecules in the ASU (which has been chosen to lie along the filament). The lower the values of |**c**|/molecule, the tighter the packing along the filament axis (Table S4).

From these data alone, it remains unclear if these filaments are biologically relevant, however, two possible effects are apparent. The first is simply that filaments provides a means of organization by clustering together light harvesting units of a single type. The second is that by creating a filament, an organism effectively brings chromophores from neighboring proteins as close as they would be within a single protein thereby increasing excitation energy transfer rates (Supplementary Fig. 2c). Some chromophores between complexes in a filament are much closer than those intra-protein. Distances between the center of each chromophore in all structures have been calculated (Supplementary Fig. 2de), along with those of adjacent molecules in *Ha*PE555 filaments (Fig. 2c). Creating a filament also generates spatial order between chromophores of neighboring PBPs. The alignment of dipoles between chromophores maximizes the FRET rate between them and the size of the cluster of chromophores that are coherent with one another.

We note that this filament arrangement in not general for *open* form PBPs as no other *open* form PBP structure to date has been observed to form filaments in this manner and it is unknown if filaments form *in vivo*. Additionally, the crystal structure of *Ha*PE560 does not display filaments. Here the crystal is formed from sheets with two $(\alpha\beta)_2$ molecules in the ASU.

To explore the possibility of filaments in *open* forms other than *Ha*PE555, we generated pseudo-filaments based on *Ha*PE555 structures by performing a least squares structural alignment on multiple copies of the *open* form of interest (Supplementary Fig. 10). The comparison of all *open* form pseudo-filaments showed that the few residues preceding the β strand S2 in each α chain are critical to the formation of filaments as, in the case of *Ha*PE560, this caused a steric clash with helix hFb of the adjacent molecule in the filament (next to the β-82 chromophore). In this region, *Ha*PE560 forms a single turn helix that protrudes from the surface (Supplementary Fig. 10, magnified view on right, *Ha*PE560 in purple) whereas *Hv*PC612 (red) and *Hp*PC577 (cyan) have a smaller protrusion which closely follows the

adjacent N-terminal end of helix hY (Supplementary Fig. 10, magnified view, helix on lower right) with a greater capacity for the formation of filaments. *Ha*PE555 (Supplementary Fig. 10, green) does neither, instead following closely along the β strand S2 (which is likely why we only see tight filaments in the crystals of *Ha*PE555).

Supplementary Note 4 —*L1* loop insertion in *Hemiselmis* α subunits between β strand *S2* and the α helix

Multiple sequence alignment of *Hemiselmis* α subunit sequences from transcriptomes has identified two clusters of sequences that possess an insertion between β strand *S2* and the α helix (Figs 1a, 2f and Supplementary Fig. 7). Ten of these sequences correspond to the *open-braced* structure described in the main manuscript (Supplementary Fig. 7 labelled canonical *open-braced*, OB +1 and OB +3). In these sequences, the *L1* loop insertion starts after an anchoring aromatic residue (tyrosine or phenylalanine) after β strand *S2* and terminates at an extra N-terminal turn on the α helix (RIG motif). The *L1 open-braced* motif is:

$$G\text{-}x\text{-}x\text{-}G\text{-}x_{1\text{-}3}\text{-}D\text{-}R$$

Ten sequences conform with this motif (clusters labelled canonical *open-braced*, OB +1 and OB +3). We note that prior to the N-terminal glycine, there is a conserved aromatic residue (phenylalanine or tyrosine) which anchors the *L1* loop.

Furthermore, there are additional sequences that contain a similar, but distinct, insertion in the same position (Supplementary Fig. 7 clusters labelled OB -2 and OB -4). These sequences come from the same organisms which contain the *open-braced* form: *H. andersenii*, *H. rufescens* and *H. tepida* each containing two sequences with these shorter insertions. Most sequences in this cluster (bar *Hr*α^{O} in cluster OB -4 from *H. rufescens*) contain the motif:

$$G\text{-}x_{1\text{-}2}\text{-}G\text{-}x\text{-}G$$

We note that one of the intact PBPs identified by mass spectrometry contains two of these sequences: *Ha*α^{O}_{3} and *Ha*α^{O}_{4} (Fig. 1a and 1e) and it belongs to peak XA in Fig. 1b. The structure of the *L1* insertion in this protein will have to await further investigations.

The alignment of all *open* form and *open-braced* form sequences highlight another conserved feature. At the end of the α helix, there is a conserved Asn-Tyr motif (Supplementary Fig. 7). This aromatic residue marks the end of the α helix, anchoring the subsequent C-terminal loop. Finally, we note that two sequences in Supplementary Fig. 7 contain a single residue insertion just prior to the characteristic Asp insertion two residues before the cysteine covalent chromophore attachment site (Supplementary Fig. 6, arrow just after β strand *S1*). Given that the Asp insertion is responsible for the transition between the *open* and *closed* quaternary structure, it is unclear as to what this extra insertion may create in terms of protein structure.

Supplementary Note 5 —Chromophore geometry and energy transfer relevant parameters

The energetic properties of each chromophore are determined by its identity, geometry, and local electrostatic environment [1]. *Ha*PE555 and *Ha*PE560 have identical chromophores (DBV on β50/61 and PEB on all other sites) whereas *Ha*PE645 differs on two points (DBV on the α subunits and the single PCB on the β82 site in the $\alpha_2\beta$ protomer; Supplementary Table 4). The geometry of the three β subunit chromophores (save the β82 of the $\alpha_2\beta$ protomer of *Ha*PE645) is largely identical between these structures within the protein matrix given they all have the same β subunit.

The key differences in chromophore geometry are those for β82 in *Ha*PE645 and the α chromophore in *Ha*PE560, both discussed in the main text, with subtler differences revealed when comparing the chromophore dihedral angles (Supplementary Fig. 6). For the α chromophore, the dihedral angles between pyrrole rings A and B partition into *closed* and *open* form clusters (Supplementary Fig. 6a-c) where the major change is due to the insertion of the aspartic acid in the sequence just before the cysteine residue covalently attached to the chromophore. In the *open-braced* form, the *L1* loop shifts and rotates the chromophore (Fig. 2de) which is reflected in the dihedral plot (Supplementary Fig. 6c). It is likely that the changes in the α chromophore are responsible for the spectral shift between *Ha*PE555 and *Ha*PE560.

For the *closed* form *Ha*PE645, the key difference is the switching of chromophores attached to cysteine β82. The geometry of the PCB chromophore attached to Cys-β82 on the $\alpha_S\beta$ is similar to that observed for *Cs*PC645 from *Chroomonas* sp (Fig. 3de). In terms of chromophore dihedral angles for pyrrole rings C and D, this chromophore forms a cluster with the two β82 chromophores from *Cs*PC645 (Supplementary Fig. 6de).

The most prominent difference between the protein matrices themselves is between the *closed* form *Ha*PE645 and the two *open* forms. In *closed* forms, the β50/61 chromophores from opposite β subunits are brought into van der Waals contact (Supplementary Fig. 2e, circled in lower panel), creating a strongly coupled quantum system, which is expected to broaden the spectrum. Strikingly, however, the shape of the main absorption peak is much the same between all three proteins (Fig. 1c; apart from the blue shift in *Ha*PE555 compared to the other two proteins). It is unclear why so little spectral broadening is observed for the *closed* form *Ha*PE645. As for the purpose of *closed* forms then, there is some suggestion that the tight binding of the central chromophores contributes to the enhanced excitation energy transfer rate (by a factor of x2 [2]), which may be a benefit of having *closed* form as the terminal acceptor of the soluble antenna and adaptor to the integral membrane systems.

Supplementary Note 6 —Mechanisms for chromophore alterations in *Ha*PE645

The asymmetry in the identity of the β82 chromophore, with PEB attached to $\alpha_L\beta$ versus PCB attached to $\alpha_S\beta$, raises question as to how this asymmetry is achieved on otherwise symmetric β subunits. No such asymmetry has been observed before in cryptophyte PBPs. The current model for how chromophores are covalently attached by lyases in cryptophytes [3] is based on an earlier model for phycobilisome assembly [4]. In these models, lyase enzymes covalently attach linear tetrapyrrole chromophores to specific cysteine residues in the plastid stroma. Chromophore attachment is coupled to folding and assembly of mature proteins prior to transit into the thylakoid lumen. These processes may involve molecular chaperones and folding/degradation pathways that control the quality and integrity of mature light harvesting proteins. Individual lyases are likely to bind to folded and/or partially assembled subunits to recognize the correct cysteine to ensure that the correct chromophore is attached.

For the β82 chromophore, the only distinguishing signal comes from the α subunit associated that forms the αβ protomer. The β82 chromophore site lies proximal to the N-terminus of the α subunit. For *Ha*PE645, the two α subunits have distinct N-terminal sequences in the vicinity of the β82 chromophore. Thus, it is likely that the lyases responsible for loading the two distinct β82 chromophores bind to the assembled αβ protomer (or possibly the fully assembled $\alpha_1\beta.\alpha_2\beta$ complex) so as to attach the correct chromophore. It has been shown that the α subunit itself acts as a chaperone for the assembly and stability of the β subunit [5]. Finally, we note that the α_L subunit would still have a thylakoid lumenal targeting sequence extending its N-terminus while it resides in the stroma. This targeting sequence may confer a signal to the lyase.

Supplementary Note 7 —FRET efficiency for the model of the *H. andersenii* antenna sandwiched between the thylakoid membranes

A model for the *H. andersenii* antenna is presented in Fig. 4. In this model, the soluble light harvesting proteins are sandwiched between the thylakoid membranes. The proteins are close packed and lack long range mobility. The bulk of the antenna is composed of *Ha*PE555 plus *Ha*PE560, which capture the most energetic photons. The question is: what is the location of the adaptor, *Ha*PE645, that accepts energy from the bulk of the antenna and transfers it to the integral membrane photosystems. To understand the constraints on the location of the adaptor, we use the model to calculate the effect of adaptor location with respect to the membrane on the efficiency of light harvesting.

FRET efficiencies were calculated analytically using a model that assumes all energy captured by the soluble PBP antenna eventually reaches the adaptor, *Ha*PE645, from which it is then transferred via FRET to the integral membrane photosystem, PS-II or others. The model begins with the FRET efficiency equations [6] where k_{ET} is the sum of FRET rates to all acceptors and k_F is the rate of fluorescence. k_{ET} itself is given by the equation below where R_0 is the Förster distance and r_i is the distance to the ith acceptor. It is assumed that R_0 is the same for all donor-acceptor pairs (*Ha*PE645—PS-II/Others).

$$E = \frac{k_{ET}}{k_{ET} + k_F}$$

$$k_{ET} = k_F \sum_i \left(\frac{R_0}{r_i}\right)^6$$

$$E = \frac{1}{1 + \left(\sum_i \left(\frac{R_0}{r_i}\right)^6\right)^{-1}}$$

The approximation in this model begins by assuming that *Ha*PE645 has a membrane acceptor directly above and below it. This leads to the equation below.

$$E = \frac{1}{1 + \left(R_0{}^6 \left(\frac{1}{(z-L)^6} + \frac{1}{(z+L)^6}\right)\right)^{-1}}$$

The equation for FRET efficiency as a function of position in the thylakoid (z) is given by the equation above where L is the half-width of the thylakoid lumenal space (distance between the bounding membranes).

The FRET efficiency, $E(z)$, was plotted for a range of values of R_0 and L (Supplementary Fig. 9). The width of the lumen (2L) is also taken to be either 12.7 nm

(Supplementary Fig. 9a) or 25 nm (Supplementary Fig. 9b) and R_0 is taken to be in a range of 3 - 6 nm [7,8]. From the plot, it can be seen that efficiency drops of quite rapidly and for maximal efficiency, the *Ha*PE645 should be placed near or tethered to the membrane.

Supplementary Note 8 —Model including filaments

The crystal structures of *Ha*PE555 indicate that some cryptophyte PBPs have a propensity to form filaments. It is possible that filament formation occurs in the biological antenna allowing for segregation of different components (to keep the *Ha*PE645 near the membrane). Another possibility is that order and regulation within the thylakoid lumen is generated by tethering the adaptor through another protein or electrostatically as a variety of isoelectric points is observed in the phycobiliproteins of other cryptophyte species [9-12]. Regulation could be triggered by a pH decrease in the thylakoid lumen from increased photosynthesis, releasing tethered *Ha*PE645 from the membrane to slow energy transfer and release oxidative stress.

In a model that includes filaments (Supplementary Fig. 12), light capture by cryptophytes is likely to proceed as follows. The model begins with the illumination of a cryptophyte by sunlight. A single solar photon with a particular wavelength (or range) sends all chromophores excitable by this wavelength into a quantum superposition. On a sub-femtosecond time scale, this quantum superposition collapses and the photon sends a chromophore or small coherent cluster of chromophores in a superposition to an excited state. Given the large fraction of *Ha*PE555 in the antenna, the excitation most likely exists as a coherent cluster on *Ha*PE555. This coherent cluster explores interactions with neighboring chromophores and the surrounding protein, expanding and/or localizing the cluster, respectively. The excitation may transfer to other chromophores/clusters incoherently via FRET along its sojourn. The coherent cluster present on *Ha*PE555 is likely to be only 2-3 chromophores in size. As the energy landscape of the chromophores of *Ha*PE555, or indeed filaments composed of *Ha*PE555, is roughly flat (because PEB and DBV have similar excitation peaks), the coherent cluster is free to diffuse along this filament. This is also the case for free *Ha*PE560 complexes. At some stage after travelling up to tens of nanometers, the coherent cluster interacts with a nearby *Ha*PE645 onto which it transfers. Once the excitation is present on *Ha*PE645 it migrates around the protein exploring the single quantum structure formed by the central pair of doubly-linked DBV β50/61 chromophores. From this position the excitation can then interact with and migrate to the single β82 PCB. This, now lower energy excitation, can interact with the integral membrane light harvesting complexes comprised mainly of carotenoids and chlorophylls, to which it will transfer (presumably by incoherent FRET) and migrate finally to the special pair of chlorophylls in the photosystem where ultimately, charge separation occurs. This whole process must take place on a nanosecond timescale otherwise the excitation will escape via fluorescence [13].

Supplementary Note 9 —History: The path to dissecting the cryptophyte antenna

Evidence for a multi-component antenna is present in early papers describing the soluble light harvesting systems of cryptophytes mainly expressing phycoerythrins. Early absorption spectra of aqueous extracts show that although the soluble antenna protein have predominant maxima around 545nm or 560-568 nm, they have smaller peaks or shoulders around 600-650nm that are somewhat variable. These studies include: *Cryptomonas ovata* [14-16], *Sennia* sp (*Hemiselmis parvula*?) [17], *Rhodomonas* CS24 [18], and *Cryptomonas acuta* [19]. Regarding *Rhodomonas* CS24, Martin and Hiller (1987) stated: "It is suggested that one or more of the α subunits of this phycoerythrin may provide the intermediate components necessary for energy transfer to chlorophyll a." [18]. It is likely that some of this wisdom was lost as protein preparations improved and laboratories focused on the most abundant components.

In addition to the spectroscopic evidence, there was abundant evidence from isoelectric focusing studies that each cryptophyte expressed multiple α subunits with different pIs. These include: *Cryptomonas* sp [20], *Cryptomonas maculata* [9], *Cryptomonas ovata* [12], *Rhodomonas* [12], *Rhodomonas* CS24 [21], *Chroomonas* [10,22] and *Hemiselmis virescens* [11].

Supplementary Note 10 —Evolutionary perspective and α sequences from other *Hemiselmids*

What is curious is why there is such a diversity of α subunit sequences in *H. andersenii*, yet most of the light harvesting appears to be done by one protein and furthermore many of the other proteins that are expressed have similar spectral properties. It is possible that the levels of all of these PBPs are regulated by environmental conditions such as light color, intensity and pH of the lumen. A recent study of photoacclimation in cryptophytes has shown that the spectral properties of isolated PBPs change when the organisms are grown in spectrally altered light [23]. Given the diversity of α subunit peptides, the levels of these mature light harvesting proteins are likely to be regulated in the cell nucleus by controlling α subunit gene expression.

One thing we do not understand is whether there is a possibility of mixing-and-matching α subunits to produce a statistical mixture of PBPs – however, cells may either have mechanisms to prevent this (such as specific chaperones) or such mixing may simply not occur due to steric selectivity. In the process of PBP maturation, it is likely that αβ protomers fold and assemble independently, and then bind to other αβ protomers rather than folding concurrently as a full set of four chains [5,24]. As such, it may be that molecular recognition between αβ protomers selects for specific pairing from the surface generated by the α subunit resulting in the well-defined mature proteins that we have observed in crystal structures.

One further observation is the diversity of α subunits in cryptophyte antennas compared to the shear lack of sequence diversity in β subunits across species [25]; the progenitor α subunit genes were relocated to the nucleus from the red algal nucleus following secondary endosymbiosis where progeny α subunits proliferated while the β subunit gene remained a single, chloroplastic gene. In fact, nuclear genes have roughly 3x the mutation rate of plastid genes in *H. andersenii* [26]. The implication for evolution here may be that α subunits are free to diversify and roam the sequence space, finding new possible niches while the β subunits remain relatively constant. The diversity of α subunits is key to an apparent modularity in cryptophyte light harvesting and its potential evolution, adaptation and tunability.

In an evolutionary context, *H. andersenii* highlights the diversification of light harvesting in cryptophytes. Firstly, it presents a mixture of *open* and *closed* forms as well as the novel *open-braced* form. Evidence for the *open-braced* form (including the shorter version of the *L1* loop) has been observed in a handful of *Hemiselmis* transcriptomes (*H. andersenii*, *Hemiselmis tepida* and *Hemiselmis rufecens*) where the *L1* loop insertion is seen (Fig. 2 and Supplementary Fig. 7). This is in part due to limited data, however, it may reflect evolutionary history and a functional role for the *open-braced* form in these organisms. As noted in the main text, although the *open-braced* form is likely more stable than the *open* form, these two forms co-exist in these organisms and in fact in the lumen of the thylakoid, the *open* form remains the dominant protein by mass. Since the innovation of the *open-braced* form is observed in a handful of *Hemiselmis* species, it is possible that *H. andersenii*, *H. rufecens* and *H. tepida* represent the most recent evolutionary branch (given current data) in the cryptophytes, with the development of the *open* forms more generally arising in *Hemiselmis*. Finally, we note that two α subunit sequences from *H. rufecens* have an extra single amino acid insertion before the

aspartic acid insertion that differentiates the *open* form, forming an entirely new innovation. It is unclear what this ‘double-open’ insert is doing to the structure.

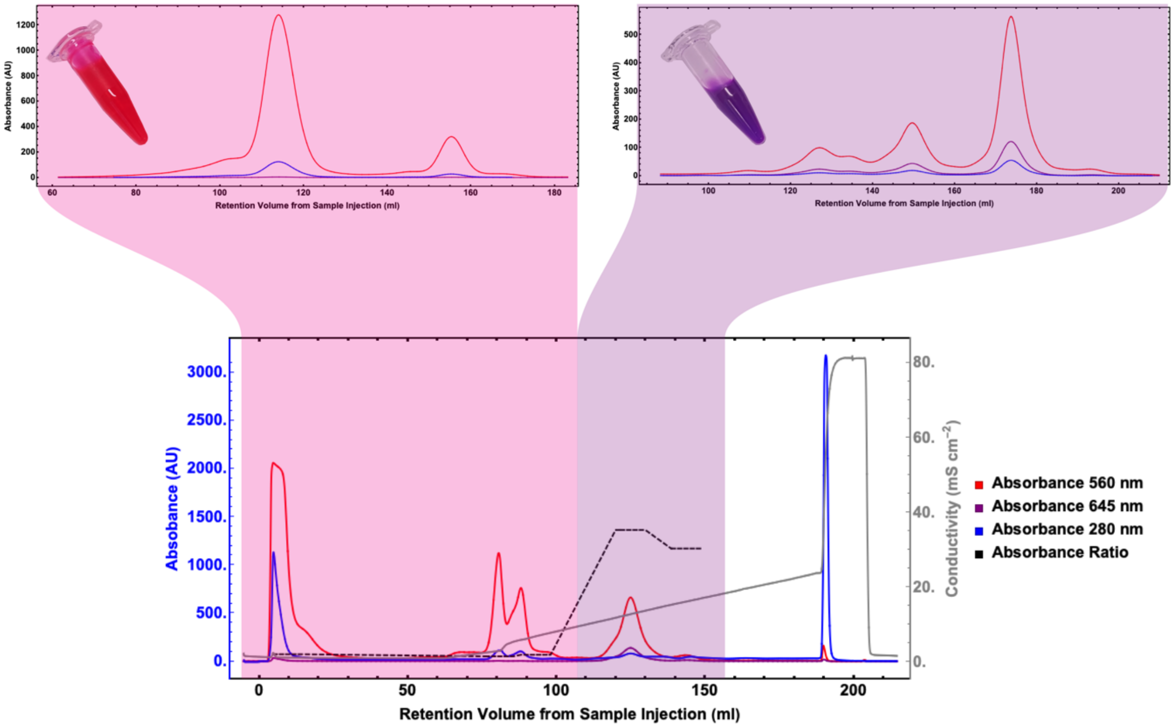

Supplementary Figure 1. Purification of soluble light harvesting protein. Lower chromatogram shows the separation of soluble light harvesting protein into pink and purple fractions using anion exchange chromatography. The two upper panels show further purification of each of these pooled fractions by cation exchange chromatography.

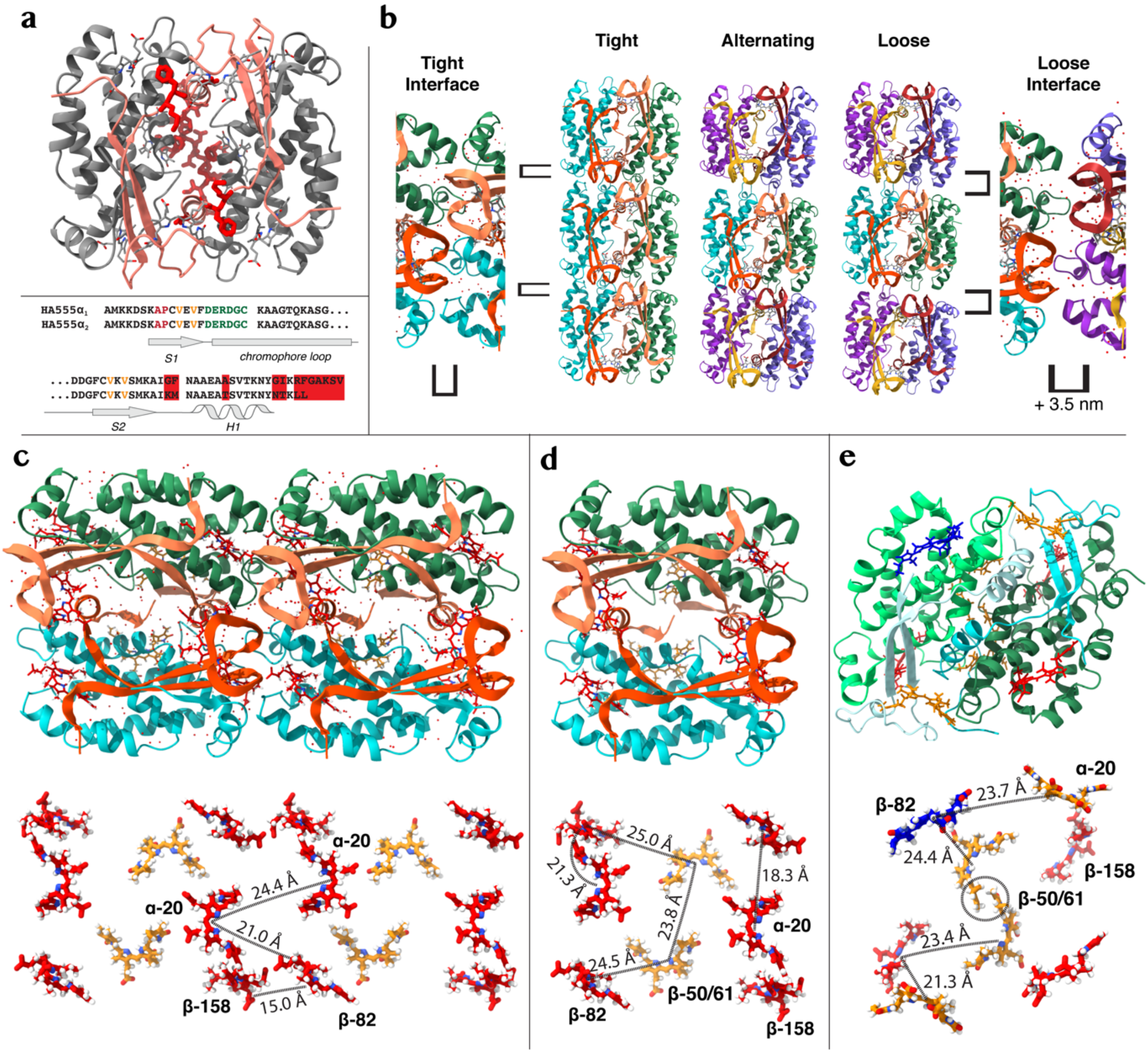

Supplementary Figure 2. The crystal structures of *Ha*PE555 are composed of sheets of filaments. a. The crystal structure of HaPE555 shows a quasi-symmetric dimer with an *open* form quaternary structure which is identical to previously published 4LMX. The two α subunits (peach ribbon) show only minor sequence differences (highlighted in red as overlapped stick figures on the structure, top, and the sequence, bottom). These minor sequence differences result in microheterogeneity in all crystal forms, hence each site is a 50:50 mix of the two distinct residues. **b.** Each crystal form of *Ha*PE555 is composed of 2D layers of *Ha*PE555 filaments that differ in the packing of the interface between monomers along the filament axis. The left most image is the tight interface while the right most image is the loose interface (each shown rotated 90° with respect to the filament images in the central panel). The

central part of the panel shows the three distinct filament types, where the left most filament shows tight interface packing, the right most filament shows loose interface packing and the central filament shows alternating tight-loose packing. The nature of the interfaces is indicated by the square brackets, where loose interface has a 3.5 Å wide layer of ordered solvent between two neighboring proteins. The presence of filaments in all crystal forms may have biological significance. **c**-**e**. Show the distances between chromophores for: **c**. the interfilament tight interface; **d**. the intramolecular distances for *Ha*PE555; and **e**. *Ha*PE645. Chromophores are shown as sticks with carbon atoms colored: red – PEB; orange – DBV; and blue – PCB. The upper panels show the complete structures that support these chromophore arrangements.

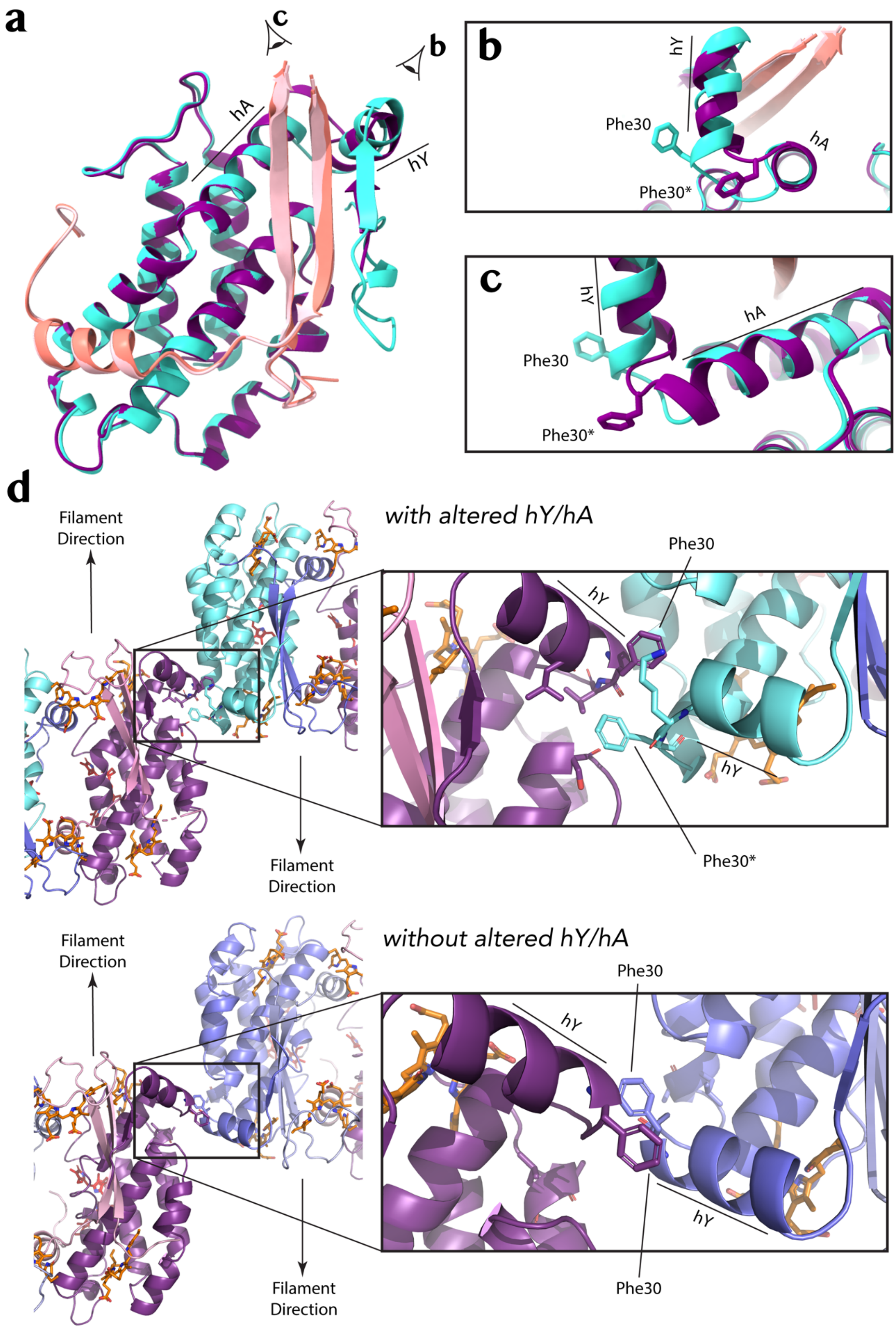

Supplementary Figure 3. Comparison of the crystal structures of *Ha*PE555A and *Ha*PE555B. a. Superposition of the structure of *Ha*PE555A (α subunit salmon, β subunit cyan) and *Ha*PE555B (α subunit light pink, β subunit purple). **b**. and **c**. show two enlargements of

the superposition from the perspectives indicated by the eye symbols in **a**. These enlargements show the differences in α helices hY and hA with the associated change in the position of Phe30 in the two structures. **d**. The structural changes in helix hY and Phe30 alter the interfilament packing in *Ha*PE555B (top) compared with *Ha*PE555A (bottom), resulting in tighter packing in the former.

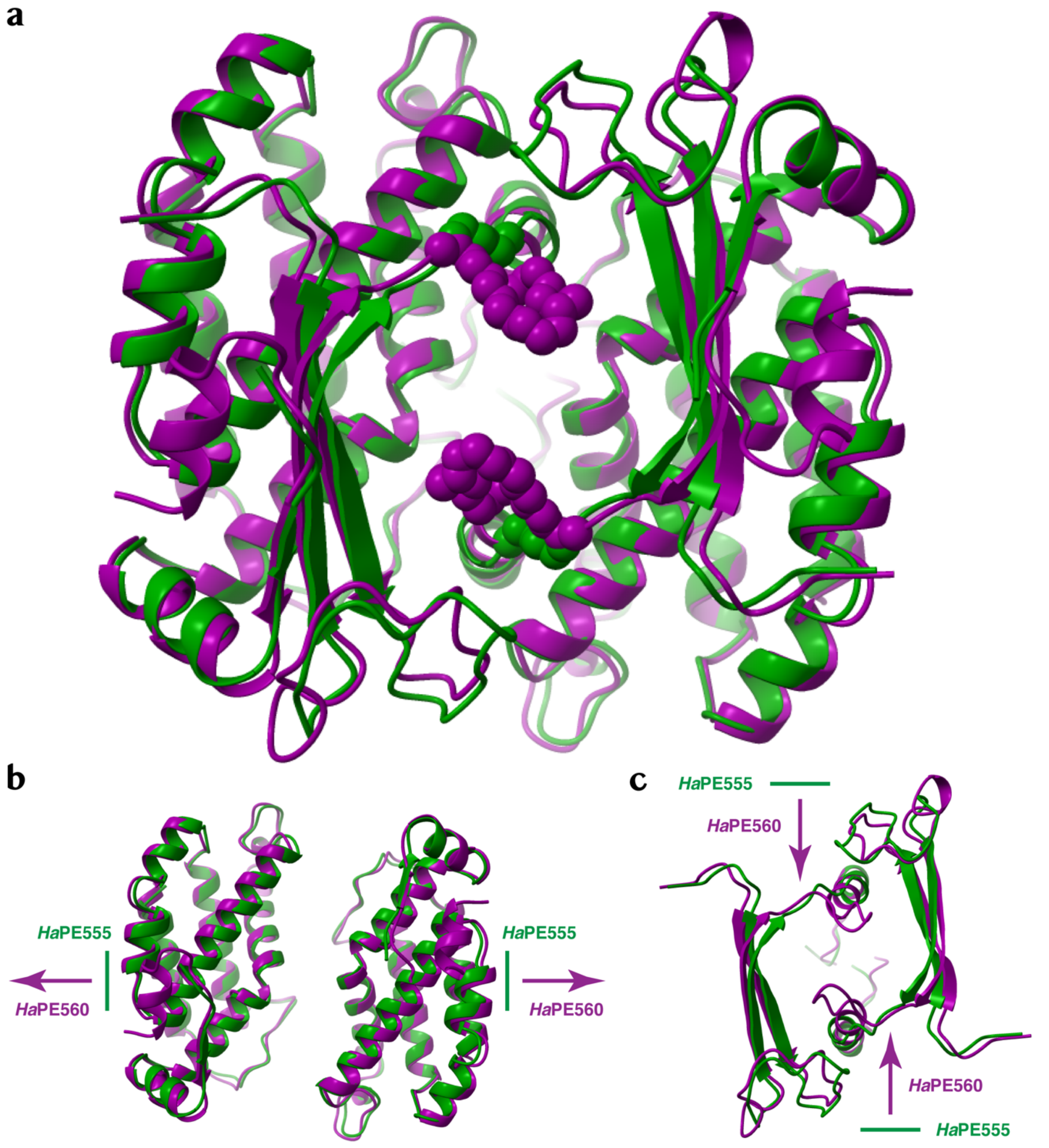

Supplementary Figure 4. Comparison of *open* and *open-braced* forms and the Poisson effect observed when comparing the *open-braced* form *Ha*PE560 with the *open* form *Ha*PE555. a. Superposition of the *open-braced* form structure of *Ha*PE560 (purple) and the *open* form *Ha*PE555 (green). The *L1* loop, and the comparable path in *Ha*PE555, is displayed as a string of spheres to highlight how the incursion begins to fill the central solvent filled hole. The Poisson effect comprises a vertical compression coupled with a horizontal expansion of *Ha*PE560 with respect to *Ha*PE555. **b**. The horizontal displacements of the *Ha*PE560 β subunits resulting in a horizontal widening of the *open-braced* form compared to the *open*

form. **c**. the inward vertical compression of the two *Ha*PE560 α subunits when compared to *Ha*PE555 complete the Poisson effect.

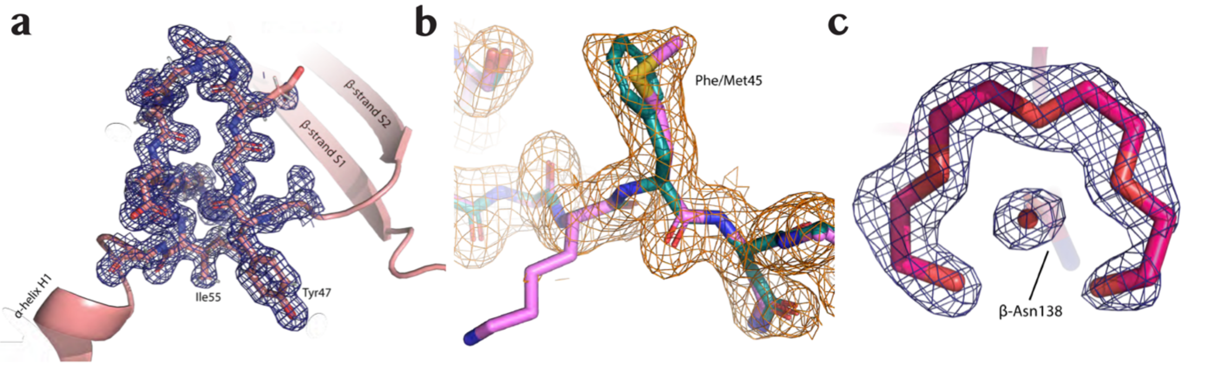

Supplementary Figure 5. Electron density for structural features of note. **a**. Electron density map for the *Ha*PE560 α subunit *L1* loop overlayed on the structure. **b**. Electron density map for *Ha*PE555 showing the microheterogeneity at Met45/Phe45 in the α subunit. **c**. PEG molecule surrounds a water molecule that is hydrogen bonded to the side chain of Asn138 in the β subunit of *Ha*PE645.

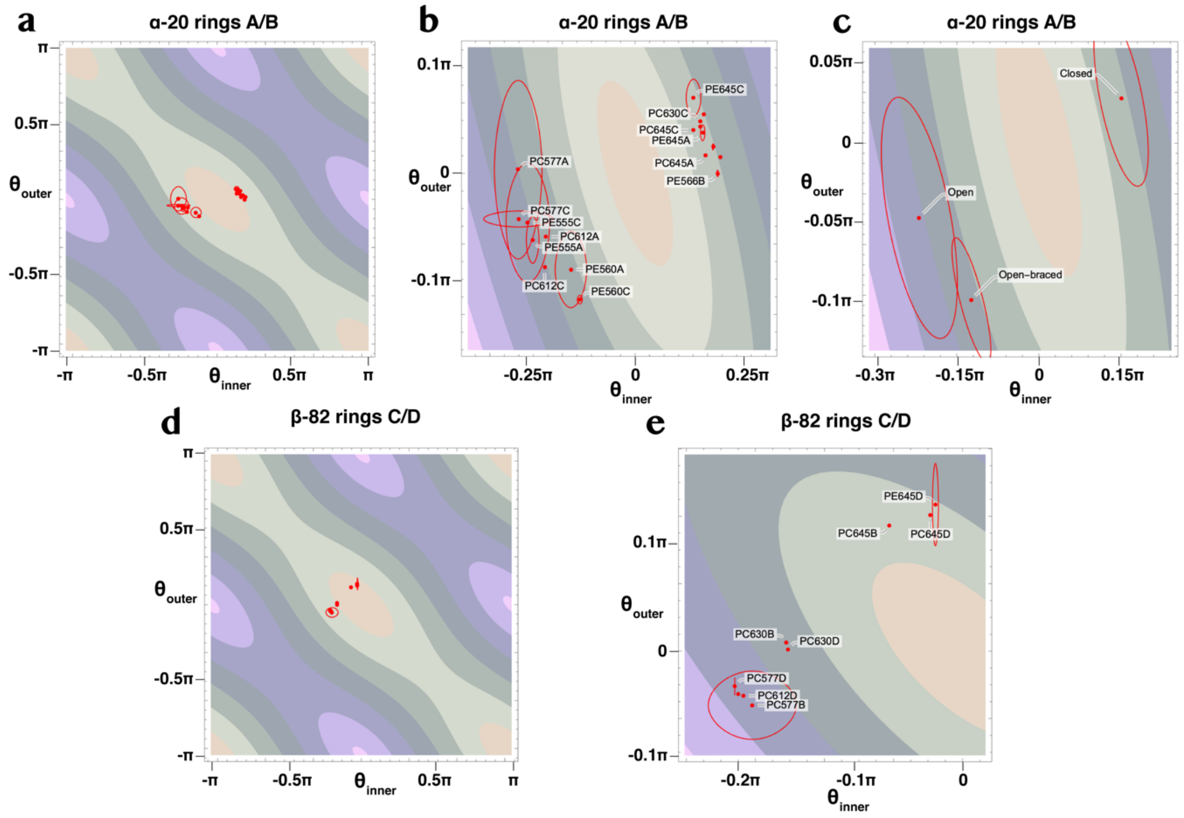

Supplementary Figure 6 Chromophore torsion angles. **a-c**. The torsion angles between pyrrole rings A and B of the α chromophores. **a**. shows the full angular range where the *open* forms are clustered on the left of center while the *closed* forms are on the right. **b**. is a close up of the same plot as **a**. **c**. here the three quaternary structures: *open*, *open-braced* and *closed* separate into three distinct regions of dihedral space. **d-e**. The torsion angles between pyrrole rings C and D of the β82 chromophore. **d**. shows the full angular range while **e**. is a close up showing the orientations of ring D in the chromophore.

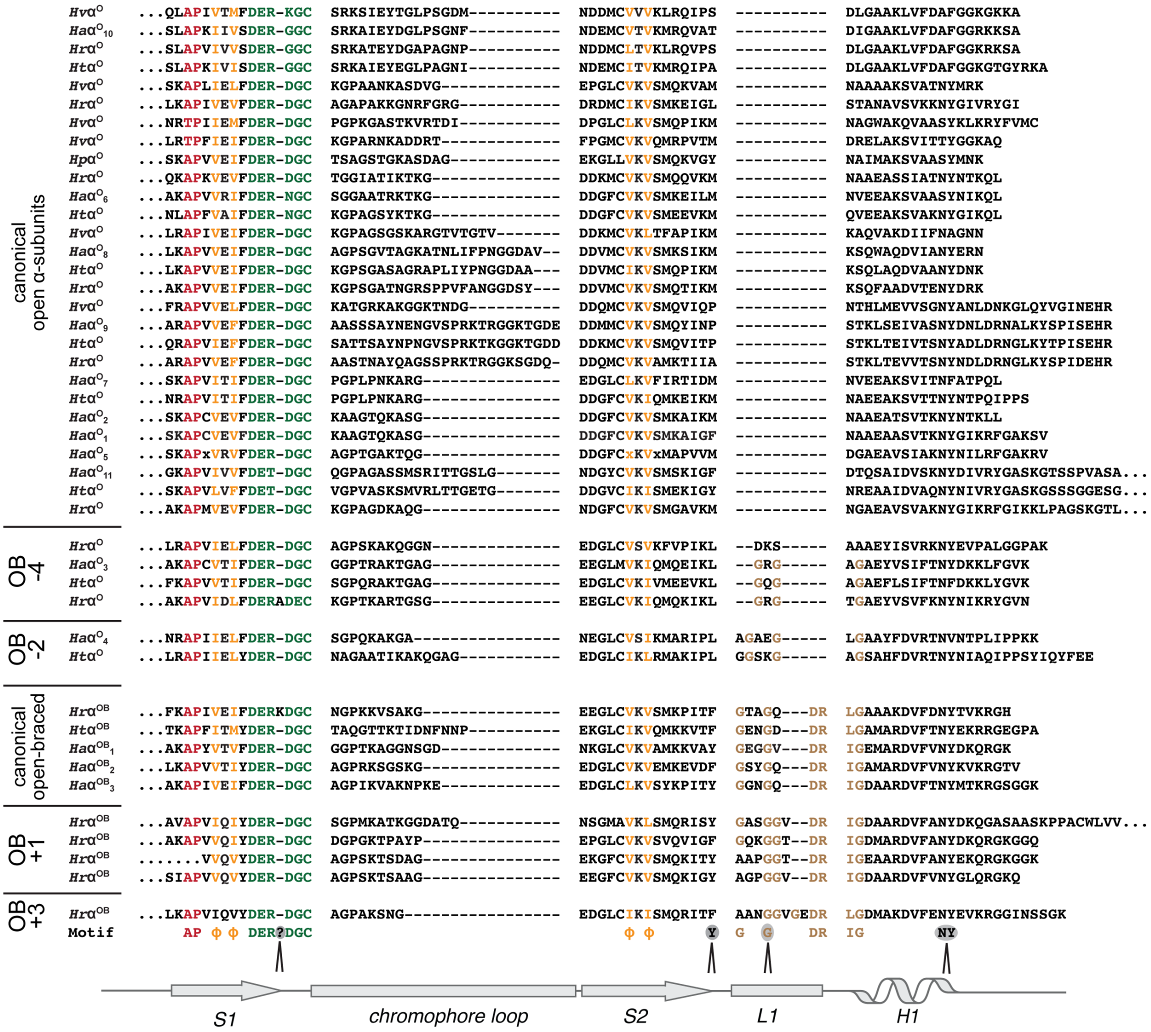

Supplementary Figure 7. Structure-based alignment of *open* form *Hemiselmis* α subunits highlighting *L1* insertions between β strand *S2* and the α helix. An alignment of all *Hemiselmis* α subunits that show the *open* form characteristic (aspartic acid insertion two residues before the chromophore binding cysteine). The top block of sequences are canonical *open* form α subunits. The lower sequence blocks show sequences that contain an insertion between β strand *S2* and the α helix *H1* (see secondary structure at the bottom). These include the *open-braced* form as defined by the crystal structure of *Ha*PE560 (corresponding to sequence *Ha*$\alpha^{OB}{}_1$). The *L1* loop insertion for the *open-braced* form starts after an anchoring aromatic residue after β strand *S2* and terminates at an extra N-terminal turn on the α helix (RIG motif). The *L1 open-braced* motif is G-x-x-G-$x_{1\text{-}3}$-D-R. Ten sequences conform with this motif (clusters labelled canonical *open-braced*, OB +1 and OB +3). Six sequences exhibit a shorter insertion at this site (clusters labelled OB -2 and OB -4). Typically, these sequences

have a G-x_{1-2}-G-x-G motif. The nature of the shorter insertion in these residues is currently unknown. The first two letters of the sequence identifiers refer to the species: *Hv* - *H. virescens*; *Ha* – *H. andersenii*; *Hr* – *H. rufescens*; *Ht* – *H. tepida*; and *Hp* – *H. pacifica*. Color coding is as per Fig. 1a in the main manuscript.

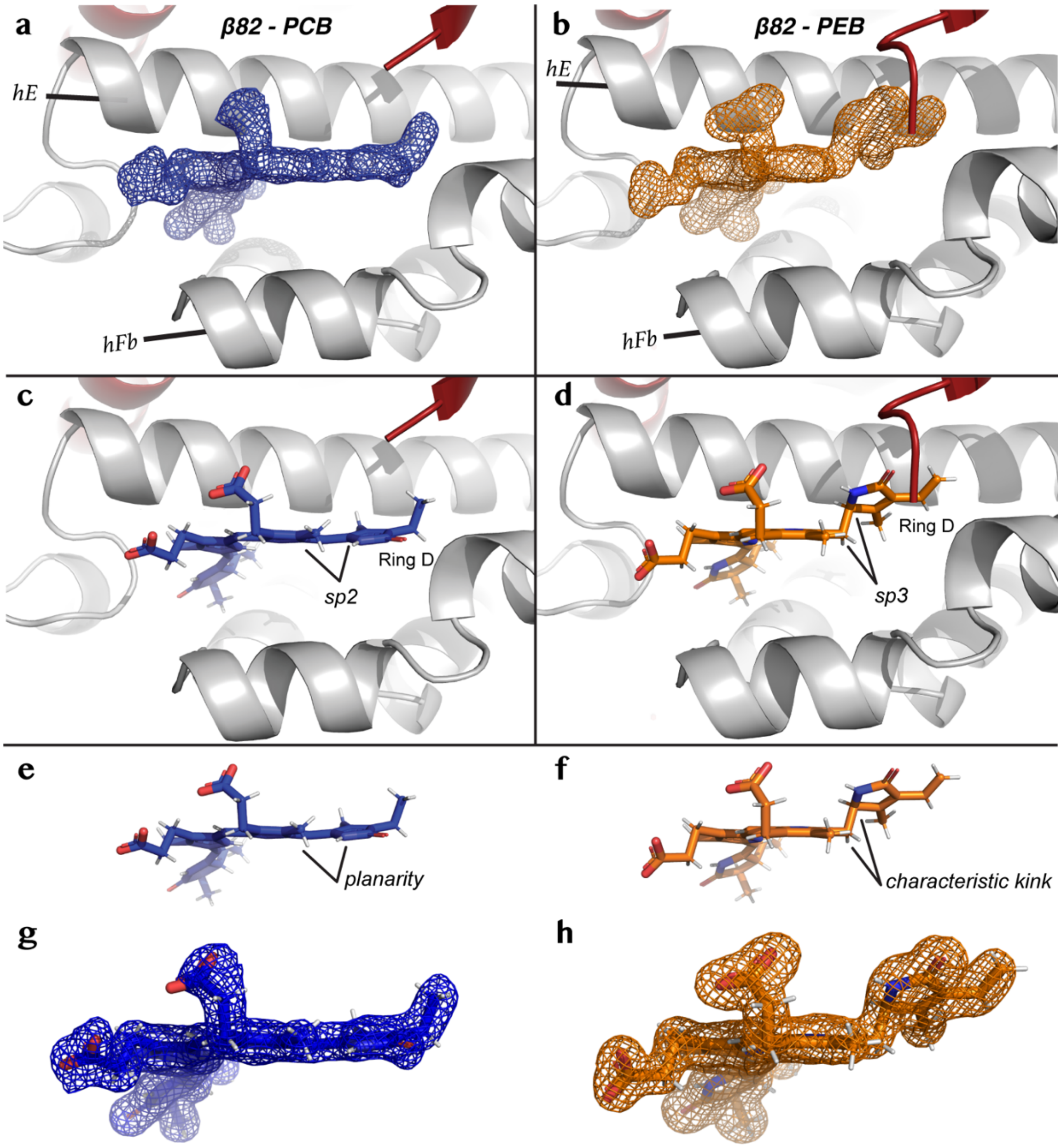

Supplementary Figure 8. Identification of chromophores attached to cysteine β82 in *Ha*PE645. Polder OMIT maps and fitted models of PCB β82 (**a, c, e** and **g)** and PEB β82 (**b, d, f** and **h)** of *Ha*PE645. **a** and **b** calculated polder OMIT maps of the asymmetric chromophore of *Ha*PE645. Note the dramatic kink induced in the PEB compared to PCB. **c** and **d** model of each chromophore within the protein matrix. The sp2 hybridisation of PCB between pyrrole rings C and D forces a planar conformation while the sp3 hybridisation of the PEB induces a kink between rings C and D. This change in geometry between the two chromophores disallows the fitting of one chromophore into the density of the other. **e** and **f** chromophores alone to highlight geometry. **g** and **h** chromophores within density and with the protein matrix removed to highlight fit.

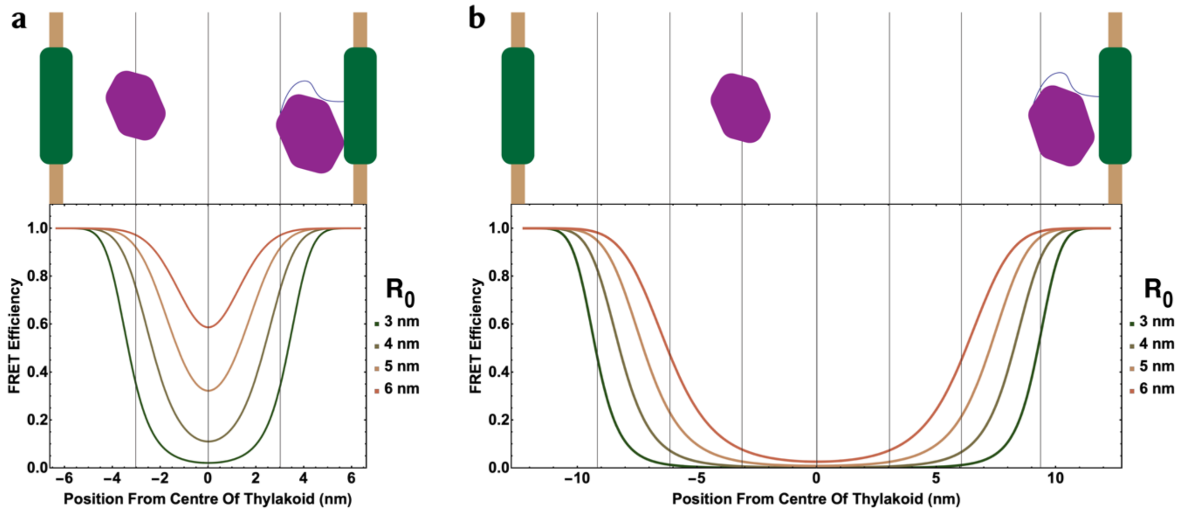

Supplementary Figure 9. Modelling the efficiency of energy transfer to the membrane systems as a function of the location of the adaptor, *Ha*PE645, within the thylakoid lumen. **a**. Plot of FRET efficiency as a function of the position of *Ha*PE645 in a 12.7 nm wide thylakoid lumen. **b**. the same calculation for a 25 nm wide lumen, representing the half the extreme width of cryptophyte thylakoid compartments. Vertical lines represent a 'protein width'.

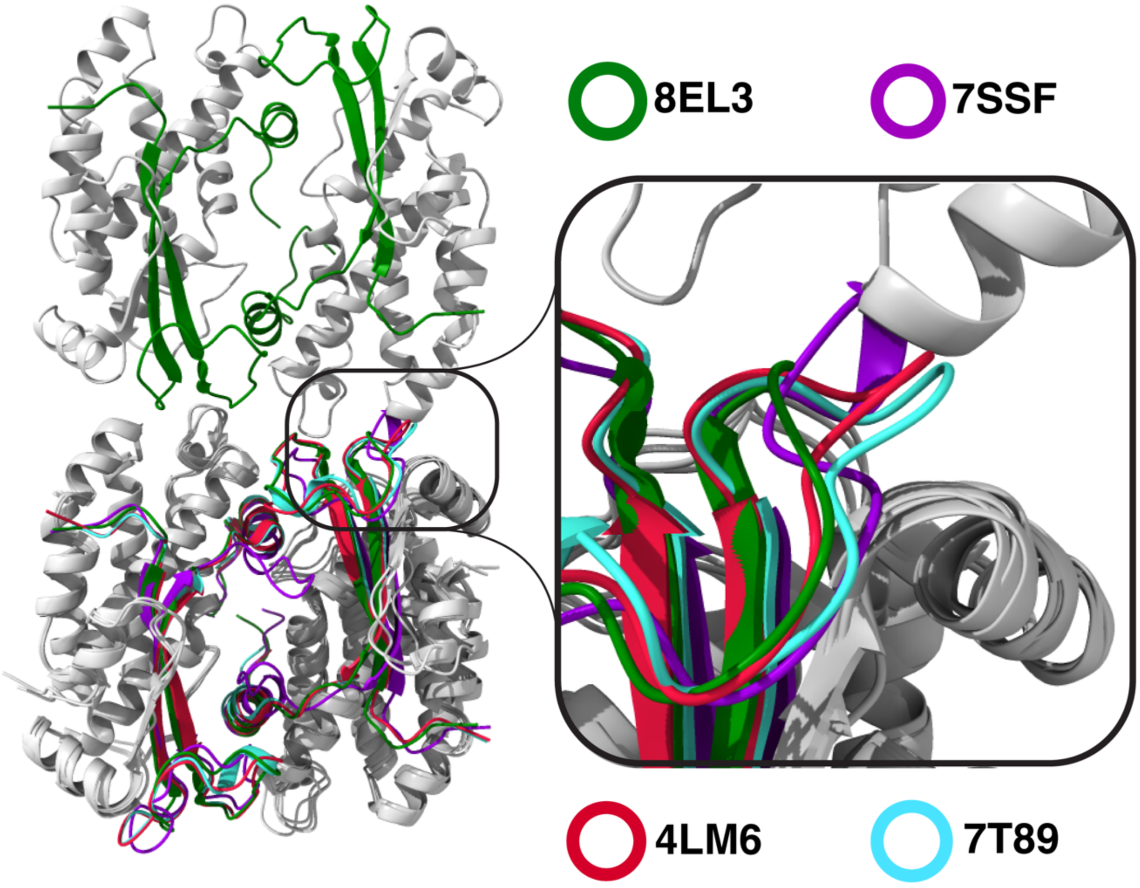

Supplementary Figure 10. Filament formation by *Ha*PE555 facilitated by α subunit chromophore loop structure. Left panel shows the filament formed by *Ha*PE555 with a loose interface (8EL3). The upper molecule is *Ha*PE555 with the α subunits shown in green and the β subunits, gray. On the lower molecule, a complete PBP, $(\alpha\beta)_2$, has been superposed for each of: *Ha*PE560 (purple, 7SSF); *Hv*PC612 (red, 4LM6); and *Hp*PC577 (cyan, 7T89). As can be seen in the magnified view (right panel), all of these overlayed structures result in a steric clash with the neighboring molecule along the filament axis. The most severe clash is for *Ha*PE560, with smaller clashes for *Hv*PC612 and *Hp*PC577. Thus, without a structural rearrangement, only *Ha*PE555 is capable of forming the filament structures seen in all of its crystal forms. Chromophores are removed from view for clarity.

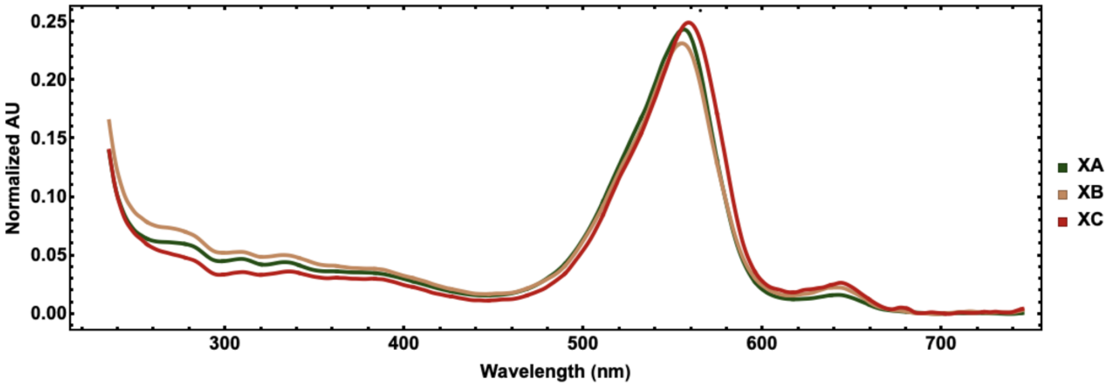

Supplementary Figure 11. Absorption spectra of unclassified peaks in chromatography. Three minor peaks in the chromatogram in Fig. 1b (left) show absorption spectra that appear to indicate that they are mixtures of different spectrotypes (see Supplementary Note 1).

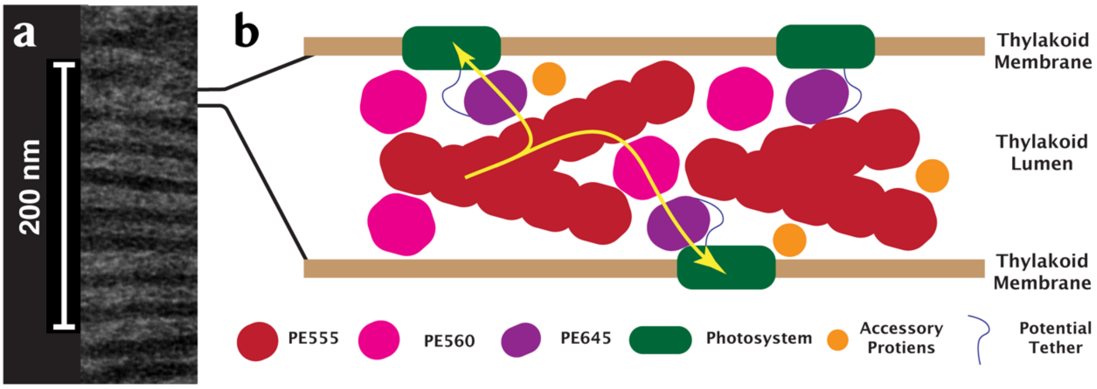

Supplementary Figure 12. Antenna model including *Ha*PE555 filaments. **a**. electron micrograph showing electron dense material (dark bands) between the thylakoid membranes. **b**. model for the light harvesting antenna that includes filaments formed by *Ha*PE555.

Supplementary Table 1. Transcriptome α subunit sequences

α subunit	Strain	Accession Code
**$HA\alpha^{O}_{1}$**	—	*From PDB 4LMX*
**$HA\alpha^{O}_{2}$**	CCMP1180	MMETSP1042-15997
**$HA\alpha^{O}_{3}$**	CCMP44	MMETSP1043-10158
**$HA\alpha^{O}_{4}$**	CCMP1180	MMETSP1042-7575
**$HA\alpha^{O}_{5}$**	CCMP441	MMETSP1043-6558
**$HA\alpha^{O}_{6}$**	CCMP439	MMETSP1041-8454
**$HA\alpha^{O}_{7}$**	CCMP439	MMETSP1041-10848
**$HA\alpha^{O}_{8}$**	CCMP644	MMETSP0043_2-9322
**$HA\alpha^{O}_{9}$**	CCMP1180	MMETSP1042-9535
**$HA\alpha^{O}_{10}$**	CCMP1180	MMETSP1042-7689
**$HA\alpha^{O}_{11}$**	CCMP644	MMETSP0043_2-10099
**$HA\ \alpha^{OB}_{1}$**	CCMP1180	MMETSP1042-7728
**$HA\alpha^{OB}_{2}$**	CCMP441	MMETSP1043-5984
**$HA\alpha^{OB}_{3}$**	CCMP1180	MMETSP1042-13440
**$HA\alpha^{C}_{1}$**	CCMP1180	MMETSP1042-4425
**$HA\alpha^{C}_{2}$**	CCMP1180	MMETSP1042-7955
**$HA\alpha^{C}_{3}$**	CCMP1180	MMETSP1042-22136
**$HA\alpha^{C}_{4}$**	CCMP441	MMETSP1043-8390
**$HA\alpha^{C}_{5}$**	CCMP441	MMETSP1043-11864
**$HA\alpha^{C}_{6}$**	CCMP441	MMETSP1043-6161
**$HA\alpha^{C}_{7}$**	CCMP644	MMETSP0043_2-17359
**$HA\alpha^{C}_{8}$**	CCMP644	MMETSP0043_2-29428

Common names of proteins and their accession codes.

Supplementary Table 2. Relative abundance of each light harvesting component by spectrotype

Gross Color Class	Percentage	Protein	% Of Gross Class	% Of Total
Pink	85 ± 1%	*Ha*PE555A	74%	63 ± 1%
		*Ha*PE560A	16%	14 ± 1%
		Others	10%	8 ± 1%
Purple	15 ± 1%	*Ha*PE645A	53%	8 ± 1%
		Others	47%	7 ± 1%

Supplementary Table 3. Filament parameters for the four *Ha*PE555 crystal structures

PDB	ASU Content (# PBPs)	|c| / molecule (Å)	Condition (+25.25% PEG3350)
8EL3	2	51.8	0.01M NaBr + 25.25% PEG3350
8EL5	2	49.7	0.2% (w/v) benzamidine HCl + 25.25% PEG3350
8EL4	1	48.3	0.01M sarcosine + 25.25% PEG3350
8EL6	1	48	23.6% PEG3350

The structures are ranked from that with the loosest interactions along the filament axis to that with the tightest interactions along the filament axis.

Supplementary Table 4. Chromophore complement for the three different spectrotypes

Protein	alpha	50	82	158
*Ha*PE555	PEB	DBV	PEB	PEB
*Ha*PE560	PEB	DBV	PEB	PEB
*Ha*PE645	DBV	DBV	PCB/PEB	PEB

Supplementary References

1 Michie, K. A. *et al.* Molecular structures reveal the origin of spectral variation in cryptophyte light harvesting antenna proteins. *Protein Sci* **32**, e4586 (2023). https://doi.org:10.1002/pro.4586

2 Jumper, C. C., van Stokkum, I. H. M., Mirkovic, T. & Scholes, G. D. Vibronic Wavepackets and Energy Transfer in Cryptophyte Light-Harvesting Complexes. *J Phys Chem B* **122**, 6328-6340 (2018). https://doi.org:10.1021/acs.jpcb.8b02629

3 Overkamp, K. E. *et al.* Chromophore composition of the phycobiliprotein Cr-PC577 from the cryptophyte Hemiselmis pacifica. *Photosynth Res* **122**, 293-304 (2014). https://doi.org:10.1007/s11120-014-0029-1

4 Anderson, L. K. & Toole, C. M. A model for early events in the assembly pathway of cyanobacterial phycobilisomes. *Mol Microbiol* **30**, 467-474 (1998).

5 Laos, A. J. *et al.* Cooperative Subunit Refolding of a Light-Harvesting Protein through a Self-Chaperone Mechanism. *Angew Chem Int Ed Engl* (2017). https://doi.org:10.1002/anie.201607921

6 May, V. & Kühn, O. *Charge and Energy Transfer Dynamics in Molecular Systems*. (Wiley, 2011).

7 Krasilnikov, P. M., Zlenko, D. V. & Stadnichuk, I. N. Rates and pathways of energy migration from the phycobilisome to the photosystem II and to the orange carotenoid protein in cyanobacteria. *FEBS Lett* **594**, 1145-1154 (2020). https://doi.org:10.1002/1873-3468.13709

8 Grabowski, J. & Gantt, E. PHOTOPHYSICAL PROPERTIES OF PHYCOBILIPROTEINS FROM PHYCOBILISOMES: FLUORESCENCE LIFETIMES, QUANTUM YIELDS, AND POLARIZATION SPECTRA. *Photochemistry and Photobiology* **28**, 39-45 (1978). https://doi.org:https://doi.org/10.1111/j.1751-1097.1978.tb06927.x

9 Morschel, E. & Wehrmeyer, W. Multiple forms of phycoerythrin-545 from Cryptomonas maculata. *Arch Microbiol* **113**, 83-89 (1977). https://doi.org:10.1007/BF00428585

10 Morschel, E. & Wehrmeyer, W. Cryptomonad biliprotein: phycocyanin-645 from a Chroomonas species. *Arch Microbiol* **105**, 153-158 (1975). https://doi.org:10.1007/BF00447130

11 Glazer, A. N. & Cohen-Bazire, G. A comparison of cryptophytan phycocyanins. *Arch Microbiol* **104**, 29-32 (1975). https://doi.org:10.1007/BF00447296

12 Brooks, C. & Gantt, E. Comparison of phycoerythrins (542, 566nm) from cryptophycean algae. *Arch Mikrobiol* **88**, 193-204 (1973). https://doi.org:10.1007/BF00421845

13 Rathbone, H. W., Davis, J. A. & Curmi, P. M. in *Photosynthesis in Algae Advances in Photosynthesis and Respiration* (eds A.W. Larkum, J. A. Raven, & A. Grossman) (Springer Verlag, 2020).

14 MacColl, R., Berns, D. S. & Gibbons, O. Characterization cryptomonad phycoerythrin and phycocyanin. *Arch Biochem Biophys* **177**, 265-275 (1976). https://doi.org:10.1016/0003-9861(76)90436-7

15 Haxo, F. T. & Fork, D. C. Photosynthetically active accessory pigments of cryptomonads. *Nature* **184**, 1051-1052 (1959). https://doi.org:10.1038/1841051a0
16 Allen, M. B., Dougherty, E. C. & Mc, L. J. Chromoprotein pigments of some cryptomonad flagellates. *Nature* **184**, 1047-1049 (1959). https://doi.org:10.1038/1841047a0
17 O'hEocha, C. & Raftery, M. Phycoerythrins and phycocyanins of cryptomonads. *Nature* **184**, 1049-1051 (1959). https://doi.org:10.1038/1841049a0
18 Martin, C. D. & Hiller, R. G. Subunits and chromophores of a type I phycoerythrin from a Chroomonas sp. (Cryptophyceae). *Biochimica et Biophysica Acta (BBA) - General Subjects* **923**, 88-97 (1987). https://doi.org:https://doi.org/10.1016/0304-4165(87)90130-9
19 Hill, D. R. A. & Rowan, K. S. The biliproteins of the Cryptophyceae. *Phycologia* **28**, 455-463 (1989). https://doi.org:10.2216/i0031-8884-28-4-455.1
20 Glazer, A. N., Cohen-Bazire, G. & Stanier, R. Y. Characterization of phycoerythrin from a Cryptomonas sp. *Arch Mikrobiol* **80**, 1-18 (1971). https://doi.org:10.1007/BF00410574
21 Hiller, R. G. & Martin, C. D. Multiple forms of a type I phycoerythrin from a Chroomonas sp. (Cryptophyceae) varying in subunit composition. *Biochim Biophys Acta* **923**, 98-102 (1987).
22 MacColl, R., Habig, W. & Berns, D. S. Characterization of phycocyanin from Chromonas species. *J Biol Chem* **248**, 7080-7086 (1973).
23 Spangler, L. C., Yu, M., Jeffrey, P. D. & Scholes, G. D. Controllable Phycobilin Modification: An Alternative Photoacclimation Response in Cryptophyte Algae. *ACS Cent Sci* **8**, 340-350 (2022). https://doi.org:10.1021/acscentsci.1c01209
24 Broughton, M. J., Howe, C. J. & Hiller, R. G. Distinctive organization of genes for light-harvesting proteins in the cryptophyte alga Rhodomonas. *Gene* **369**, 72-79 (2006). https://doi.org:S0378-1119(05)00638-4 [pii]
10.1016/j.gene.2005.10.026
25 Rathbone, H. W., Michie, K. A., Landsberg, M. J., Green, B. R. & Curmi, P. M. G. Scaffolding proteins guide the evolution of algal light harvesting antennas. *Nat Commun* **12**, 1890 (2021). https://doi.org:10.1038/s41467-021-22128-w
26 Grisdale, C. J., Smith, D. R. & Archibald, J. M. Relative Mutation Rates in Nucleomorph-Bearing Algae. *Genome Biol Evol* **11**, 1045-1053 (2019). https://doi.org:10.1093/gbe/evz056
