# Supplementary material for: Molecular dissection of the soluble photosynthetic antenna from the cryptophyte alga Hemiselmis andersenii
Source: Commun Biol. 2023 Nov 13;6:1158. doi: 10.1038/s42003-023-05508-4 (PMC10643455; doi:10.1038/s42003-023-05508-4)
Supplement: Supplementary file 3 — Description of Additional Supplementary Data [file 42003_2023_5508_MOESM3_ESM.docx]

**Description of Additional Supplementary Files**

**File name:** Supplementary Data 1

**Description:** Source data behind the chromatograms in Figure 1b.

**File name:** Supplementary Data 2

**Description:** Source data behind absorption spectra in Figures 1c and 4d-f. Columns are the absorption values at the wavelength given in the column header. Rows are the individual proteins labelled in the first column. Spectra are grouped as they are in the figures.

**File name:** Supplementary Data 3

**Description:** Source data behind fluorescence spectra in Figure 4e-f. Columns are labelled with fluorescence wavelength and rows with the protein.

**File name:** Supplementary Data 4

**Description:** Source data behind the excitation-emission maps shown in Figure 1f and Figure 4f. The columns are labelled with the excitation wavelength while the rows are labelled with the emission wavelength. The three excitation-emission maps, one for HaPE555, HaPE560 and HaPE645, are stored as separate sheets in this file.
