# Supplementary material for: Molecular dissection of the soluble photosynthetic antenna from the cryptophyte alga Hemiselmis andersenii
Source: Commun Biol. 2023 Nov 13;6:1158. doi: 10.1038/s42003-023-05508-4 (PMC10643455; doi:10.1038/s42003-023-05508-4)
Supplement: Supplementary file 8 — Life sciences reporting summary [file 42003_2023_5508_MOESM8_ESM.pdf]

nature portfolio

Corresponding author(s): Paul Curmi

Last updated by author(s): Sep 21, 2023

Reporting Summary

Nature Portfolio wishes to improve the reproducibility of the work that we publish. This form provides structure for consistency and transparency in reporting. For further information on Nature Portfolio policies, see our Editorial Policies and the Editorial Policy Checklist.

Statistics

For all statistical analyses, confirm that the following items are present in the figure legend, table legend, main text, or Methods section.

n/a	Confirmed	
☒	☐	The exact sample size (*n*) for each experimental group/condition, given as a discrete number and unit of measurement
☒	☐	A statement on whether measurements were taken from distinct samples or whether the same sample was measured repeatedly
☒	☐	The statistical test(s) used AND whether they are one- or two-sided<br>*Only common tests should be described solely by name; describe more complex techniques in the Methods section.*
☒	☐	A description of all covariates tested
☒	☐	A description of any assumptions or corrections, such as tests of normality and adjustment for multiple comparisons
☒	☐	A full description of the statistical parameters including central tendency (e.g. means) or other basic estimates (e.g. regression coefficient) AND variation (e.g. standard deviation) or associated estimates of uncertainty (e.g. confidence intervals)
☒	☐	For null hypothesis testing, the test statistic (e.g. *F*, *t*, *r*) with confidence intervals, effect sizes, degrees of freedom and *P* value noted<br>*Give P values as exact values whenever suitable.*
☒	☐	For Bayesian analysis, information on the choice of priors and Markov chain Monte Carlo settings
☒	☐	For hierarchical and complex designs, identification of the appropriate level for tests and full reporting of outcomes
☒	☐	Estimates of effect sizes (e.g. Cohen's *d*, Pearson's *r*), indicating how they were calculated

Our web collection on statistics for biologists contains articles on many of the points above.

Software and code

Policy information about availability of computer code

Data collection	Blu-Ice interface at the Australian Synchrotron.
Data analysis	ChimeraX 1.1; PyMol 2.3.4; Phenix 1.18; CCP4; Coot 0.8.9.2; Mathematica 12.0.0.0; PsiBLAST; MAFFT; ClustalW; MUSCLE

For manuscripts utilizing custom algorithms or software that are central to the research but not yet described in published literature, software must be made available to editors and reviewers. We strongly encourage code deposition in a community repository (e.g. GitHub). See the Nature Portfolio guidelines for submitting code & software for further information.

Data

Policy information about availability of data

All manuscripts must include a data availability statement. This statement should provide the following information, where applicable:

- Accession codes, unique identifiers, or web links for publicly available datasets
- A description of any restrictions on data availability
- For clinical datasets or third party data, please ensure that the statement adheres to our policy

Atomic coordinates for models generated in this work have been deposited with the Protein Data Bank with accession codes for: 8EL3, 8EL4 and 8EL5 - the filament containing structures of HaPE555 from chromatography peak 555A; 8EL6 - the structure of HaPE555 from chromatography peak 555B; 7SSF - HaPE560; and 7SUT - HaPE645.

Human research participants

Policy information about studies involving human research participants and Sex and Gender in Research.

Reporting on sex and gender	not applicable
Population characteristics	not applicable
Recruitment	not applicable
Ethics oversight	not applicable

Note that full information on the approval of the study protocol must also be provided in the manuscript.

Field-specific reporting

Please select the one below that is the best fit for your research. If you are not sure, read the appropriate sections before making your selection.

☒ Life sciences ☐ Behavioural & social sciences ☐ Ecological, evolutionary & environmental sciences

For a reference copy of the document with all sections, see nature.com/documents/nr-reporting-summary-flat.pdf

Life sciences study design

All studies must disclose on these points even when the disclosure is negative.

Sample size	All processed data sets have been deposited with the PDB under the same accession numbers as the final structures.
Data exclusions	No data were excluded.
Replication	Multiple rounds of structural refinement have been performed with Phenix, each resulting in indistinguishable final atomic models on convergence. Regions of the protein where no clear consensus structure could be obtained due to poor map quality were not included in the final atomic models deposited with the PDB.
Randomization	Randomization of samples is not relevant because we are studying specific protein complexes.
Blinding	Blinding is not relevant because we are studying specific protein complexes.

Reporting for specific materials, systems and methods

We require information from authors about some types of materials, experimental systems and methods used in many studies. Here, indicate whether each material, system or method listed is relevant to your study. If you are not sure if a list item applies to your research, read the appropriate section before selecting a response.

Materials & experimental systems

n/a	Involved in the study	
☒	☐	Antibodies
☐	☒	Eukaryotic cell lines
☒	☐	Palaeontology and archaeology
☒	☐	Animals and other organisms
☒	☐	Clinical data
☒	☐	Dual use research of concern

Methods

n/a	Involved in the study	
☒	☐	ChIP-seq
☒	☐	Flow cytometry
☒	☐	MRI-based neuroimaging

Eukaryotic cell lines

Policy information about cell lines and Sex and Gender in Research

Cell line source(s)	Cultures of the cryptophyte alga Hemiselmis andersenii strain CCMP644 were obtained from the National Center for Marine Algae and Microbiota (Bigelow Laboratory for Ocean Sciences)
Authentication	The absorption spectra and protein sequences authenticate these cells as H. andersenii.

Mycoplasma contamination	Not applicable
Commonly misidentified lines (See ICLAC register)	Not applicable
